# Extracts from the Transactions of the Baltimore Pathological Society

**Published:** 1858-05

**Authors:** W. Chew Van Bibbeb


### *Extracts from the Transaction of the Baltimore Pathological Society.*

By W. Chew Van Bibbeb, M. D.

### *Croup.*

Dr Riggin Buckler said, on Friday evening Nov. 6th, we were called to see a little girl aged 20 months, of a delicate and feeble constitution, suffering from an attack of croup. The following is the history of the case:

For the previous ten days she has had repeated attacks of catarrh, for which her parents had dosed her with ipecac., tart. emetic, &c. On the morning of Nov. 6th her cough, which hitherto had been loose and moist, changed its character, and became dry, tight and croupal. We saw her in the evening at 8 o'clock, when she presented the following symptoms: Face flushed; skin hot and dry; pulse rapid and feeble; respiration quick and apparently embarrassed; cough frequent, dry and croupal; voice very hoarse; chest sounds clear; over both lungs diffused subcrepitant rales; throat of a deep dusky red (but perfectly free from deposit); tongue dry and coated; much restlessness; tosses herself continually about the bed. Has been vomited 4 or 5 times during the day, and had one or two loose stools. Ordered tart. ant. $\frac{1}{40}$ gr. every 3 hours; hot foot bath, &c., to be repeated 2 or 3 times during the night.

Saturday, Nov. 8, 9 o'clock A. M.—Condition much the same; has slept but little during the night; antimony has nauseated her, and vomited her once; no further action on bowels. Continue antimony; give every 4 hours a powder of ii grs. calomel, $\frac{1}{4}$ gr. ipecac., iii grs. nit. potass.; apply turpentine externally to the throat; diet light.

2 o'clock P. M.—Symptoms aggravated; breathing much embarrassed; has been vomited freely, and the bowels have been opened 3 or 4 times Examined throat, and for the first time found it completely coated with a soft white caseous deposit, easily detached. Blew into throat ʒi finely powdered alum. Copious vomiting, which cleared out the throat completely. A solution of sulph. copper was then freely applied to the fauces. This afforded her much relief, and she fell into a quiet sleep of some hours' duration.

6 o'clock P. M.—Has an increase of fever; bowels are very loose; breathing easier; no alteration in the cough or voice; both dry and hoarse. Again applied copper to the throat, and ordered antimony and the powders to be discontinued.—Take of chlorate potass. 20 grs., syr. ipecac. 20 gtts. in a dessertspoonful of infus. senega every 4 hours.

11 o'clock P. M.—It was neccessary to use the alum again to relieve the throat of the deposit

Sunday Nov. 9, 9 A. M.—Has slept part of the night; has less fever, and looks pallid; heat of skin not great; bowels very loose; has had 6 or 8 copious water stools since we last saw her; cough not improved; she seems to be much exhausted; the tonsils have still deposit upon them.—Ordered an astringent mixture for the diarrhœa; continue at longer intervals (5 or 6 hours) the chlor. potass.; milk as diet; wine whey occasionally.

12 o'clock M.—Has had but one stool since we saw her; has taken more nourishment than heretofore; the deposit in the throat has increased; used the alum again, which re moved nearly all of it. Continue treatment.

6 o'clock P. M.—No improvement in croupal symptoms; diarrhœa has been checked. Injected into larynx arg. nit. grs. xx to the ounce.—A violent fit of coughing followed, during which a piece of tough, consistent false membrane, about a half inch long and a quarter inch wide, was coughed up. Free vomiting followed, and brought away more of the deposit. This afforded much relief.

11 o'clock P. M.—She fell into a sleep soon after we left, which lasted about two hours. She has a light increase of fever; and though she seems easier, still there is not the least change in the character of her cough; voice still very hoarse.

Monday Nov. 10 o'clock A. M.—Has had some sleep during the night; her general symptoms are no worse, but the local affection is still the same; there is yet much deposit in the throat. The following course was now pursued. By the aid of several screens a small tent or chamber was constructed. This was covered with blanket, and a comfortable settee for the nurse was arranged within. Into this the child well wrapped up in a blanket, was placed along with the nurse, and by means of an apparatus consisting of a tin boiler, which could be easily and constantly heated by the ordinary fire in the room, and a tin pipe of some 6 feet in length, connected with the boiler, we were enabled to introduce into the above chamber a constant supply of vapor. Continue the chlor. potass. Milk and chicken water for nourishment.

12 o'clock M.—We found the child asleep. Her skin is moist and pleasant; breathing is decidedly better, and has lost much of its harsh, dry sound.

4 o'clock P. M.—Condition much the same as at 12 o'clock; voice is still hoarse, but the respiratory sounds, together with the cough, are more moist.

In the evening, at 11 o'clock, when we made our visit, our little patient was coughing most violently, during which she vomited, and threw off several pieces of soft, greyish false membrane. Her nurse states that shortly after our last visit in the afternoon, she noticed that the cough of the child had, as she termed it, become very rattly.

The next day, Nov. 11, 10 o'clock A. M. the nurse states that she has slept nearly all the night. Her symptoms are decidely better in all respects; she seems worn out, aud inclined to sleep; cough is less frequent, and perfectly moist and free, more catarrhal than croupy in itse character; she has no fever.

From this period, for the three succeeding days, she was kept under the moderate use of the chlor. potass, and under the tent, breathing altogether the warm, moist air.

On the 16th of November, she was playing about, apparently perfectly well, and up to the present date has had no return of the croup.

We have reported this case in full, Mr. President, and must plead as an excuse for having thus trespassed upon your time, the many points of interest that cases of this kind must necessarily offer. Recoveries from this form of croup are rare occurrences, and any one to have seen how bad a subject our patient was to withstand such an attack, would never have imagined she would have recovered. Her age, delicate constitution, health already impaired by previous sickness and treatment, together with a strong predisposition to struma, were all against a favorable prognosis.

Since the first of this year, this case makes the sixth of the kind we have met with. Three occurred among our patients, one in the practice of Dr Thos. H. Buckler, one in that of Dr. Bull, and one patient belonging to no particular doctor, but operated on by a physician of this city, and died under the knife. Of the six cases, three were males; three females; three died; three recovered; one of those that died, as already stated, fell a victim to the operation of tracheotomy. (See case reported to society by Dr. F. Donaldson.) Two of the three that died were boys; both were under two years of age; the girl was over six years. The three that recovered were all under two years of age. In every instance the false membranes could be distinctly seen in the fauces. In three of these cases the chlorate potassa was given freely from almost the commencement of the attack. In two of these "the vapor inhalation" was used, and both recovered. The other occured in the summer time, and died soon after being attacked.

It may be interesting to you to know that the three cases specially ob. served by us had been in bad health, and had suffered from catarrhal and gastric affections prior to the attack of croup, and all of them were born of parents essentially strumous. The deposit on the fauces in these cases resembled granular coagulated albumen, and was soft, friable, and unctuous to the touch.

We would especially wish to direct the society's attention to the employment of the "vapor inhalation" as an adjunct in the treatment of these cases. The benefit derived from the inhalation of vapor in the treatment of laryngeal affections in the adult, are well known to medical men. It was recommended also in the treatment of infantile croup many years ago, by Dr. Warren of Boston; but the mode advised for applying it was entirely different. Thus it was suggested to place the patient in a hot bath of water and cover all with a blanket so as to get the full benefit of the steam. This method, it will be seen at a glance, has many disadvantages. The immersion of our little patient in hot baths tends much to increase their exhaustion. And besides, to the majority of children such a routine of hot bathing would frighten them so much that it is questionable whether they derive much benefit from the treatment. We have seen the vapor bath applied in two cases, in the manner which we have just described, and which was first suggested to us last winter by Dr. John Buckler. Both of the cases were decidedly benefited by it. Under its influence we have noticed—1st. The temperature of the skin is lowered; the fever is lessened; the skin becomes moist (even when the child is wrapped in a blanket.) 2dly. The breathing is easier, and the cough is less frequent, losing its dry and rude character, and becoming moist and loose.

Dr. Archibald George read the following paper upon the anatomy and diseases of

### *The Prostate Gland.*

Notwithstanding the industry lavished upon the study of this organ by anatomists, many points in relation to it are still obscure, as we may judge from the fact that the number of lobes is still a question, many writers considering it a unique body, others holding that it consists of several. It is, however, so intimately connected with the neighboring organs as to need a few anatomical considerations before we can approach the main subject of this paper.

The prostate is then, as regards its structure, a gland of the species termed "clustered" or "en grappe," composed of granulations, from each of which is sent off an excretory duct, which unites with those of neighboring granulations, to form troncules and trunks. These open for the most part, upon the inferior surface of the prostatic urethra on either side of the veru montanum. Some terminate upon the sides, or at points more or less near the superior or inferior surface of the urethra, although none have yet been found opening immediately upon the superior wall.— The largest of them open upon the posterior part of the channels existing on either side of the veru montanum, directly in front of the internal orifice of the urethra, in a cul de sac, which sometimes offers an impediment to the entrance of a sound into the bladder In the condition of hypertrophy this cul de sac becomes quite profound, and the orifices of the ducts so enlarged that a fine bougie easily penetrates in the body of gland. The number of these ducts is from 12 to 15, and when injected with mercury are found to possess an arborescent disposition. Where they terminate they pass under the muscular fibre subjacent to the mucous coat of the urethra, and open obliquely upon the latter. The smallest troncules are lined by pavement epithelium, where a tube is formed of the diameter of $\frac{1}{20}$ of a millimetre. This becomes prismatic or cylindrical, and in the larger tubes vibratile cilia are added. In addition to the proper secretory and excretory apparatus. is an accessory tissue, intimately associated with the former, and composed of fibres of cellular tissue disposed in fascettes, and accompanied by finely granular amorphous matter. These fascetted are directed in every direction, but generally run paralled with the ducts. Through this basement structure are distributed a number of nervous fillets derived from the hypogastric plexus, a dependence of the great sympathetic. Its arteries come from the inferior vesical, middle hemorrhoid and internal pudic. The gland is surronnded by a proper envelope continuous with the structure just mentioned. Its color is tawny, reddish yellow, and creaks under the scalpel. Amussat and Civiale admit that it is composed of three lobes—two lateral and one middle (inferior of Vidal de Cassis). The latter is termed by Velpeau the pathological lobe, being, according to him, the, most common cause of retention of urine. According to Hall and Morgagni, the organ is uniqve. Sir Everard Home taught same doctrine as Amusat. Spiegel and Ledran admit but two lobes, while Serres holds that it is composed of no less than four lobes—the two internal uniting at an early age to form one—a central. Whatever be their number, it is quite certain that in the adult their union is so intimate that they cannot be easily separated, if at all—a union further strengthened by the envelope referred to, and by a layer of muscular fibres from the bladder.

*Form.*—The prostate has generally been likened to a cone flattened vertically and anti-posteriorly, the base resting behind upon the neck of the bladder, and its summit being anterior and under the urethra. This statement or comparison is incorrect; the transverse diameter is greater than the vertical, and the base is not perpendicular to the axis. Roberi describes six faces to the gland, but Sappey seems to admit but two, and gives these dimensions: inferior face, 31 millemetres; superior, 18; width at base, 40; height, 27. The dimensions vary much, however, at different ages. Thus, in the child it is quite rudimentary, and in the adult does not acquire a size in proportion to the rest of the generative apparatus. In age hypertrophy is common, indeed, seems to be the proper physiological condition of the organ in advanced life.

*Position.*—The prostate is situated in the angle formed by the rectum, where it changes its antero-posterior direction for a vertical one as it plunges towards the anus. The summits of the organ and of the angle exactly correspond. Its relations with the bas fond of the bladder are these—when the latter is empty, and the rectum voided, the base of the prostate is covered by peritoneum. At the urethral orifice of the bladder its mucous coat adheres to the gland—an adherence rendered yet greater by its continuation with the prostatic portion of the urethra. But the muscular tissue of the bladder has the most intimate connections with the prostate, for some of its longitudinal fibres arising from the summit of the bladder, embrace the lateral and superior parts of the gland, and the walls of two first portions of the urethra adjacent, before being inserted upon the pubis, while others are inserted upon the base of the gland.

*Relations with the urethra.*—The urethra, in passing through the gland is lodged much nearer the superior than than the inferior surface, there being about four-fifths of the organ under the canal. Amussat and some other writers contend that the urethra merely lies in a sort of channel in the top of the gland, but their error is, as Sappey has shown, in confounding the superior portion of the prostatic ring with the sphincter of the neck of the bladder and anterior longitudinal fibres of the mass adhering to it. When the base of the prostate is examined, two canals are seen upon it. The superior and largest is that for the neck of the bladder and urethra, while the inferior corresponds to the neck of the seminal vesicles and the ejaculatory canals. The latter is lined by a fibrous sheath sent from the prostato-perineal aponeurosis. An artery, vein, and two or three lymphatics pass through this orifice These canals are not very adherent, and may be readily followed by mercurial injection. At their point of termination in the urethra, they are separated by a portion of urethral mucus, termed the "prostatic utricle," whose size is proportionare to that of the ejaculators. Its anterior extremity communicates with the urethral cavity, by an oval orifice occupying the most prominent portion of the veru montanum. When the ejaculatory ducts are full, this orifice is closed, and when they are emptied the orifice again opens. It hence seems simply to be a provision by means of which an easy dilatation may be effected for the passage of the sperm at the moment of ejaculation. Evidently this anatomical description of the prostate is necessarily imperfect, hut my limits compel me to turn to more practical points in its consideration. The use of the prostate seems to be to give support and solidity to the organs it encircles, bearing some analogy to the iron hoops which the pump maker places upon the joints of his stocks. The neck of the bladder is enabled to sustain its form, and to bear the weight of accumulated urine above it without risk of laceration. In its strong embrace the joining of the urethra and bladder is safely accomplished, and the other organs traversing it securely protected. Its function is the secretion of a fluid destined to lubricate the urethra and to mingle with the semen. It is of a milky aspect, of oily consistency, transparent, and of little tenacity. It is composed of a serum, numerous fatty granulations, gray molecular granulations, cells of prismatic epithelium, with vibratile cilia, and frequently various concretions. To this fluid the semen owes its *whitish color.*

*B. Diseases.*—One of the most interesting topics in connection with this gland is its relation to the disease termed spermatorrhœa. Its role in this affection is most important, and not less so from the fact that it is often overlooked. To the valuable work of Lallemand we owe the first light shown upon a subject then obscure—and I believe that at the present date the principal doctrines advanced by him are placed beyond a doubt—that nocturnal pollutions are consequent upon excess of venery, masturbation, indulgence of erotic ideas and reading of filthy books, occurring with erection and sensation—which become diminished as the affection advances; that diurnal pollutions are primarily dependent upon the same causes and secondarily upon slight irritation even; sometimes a lascivious idea, or touching the genitals, with slight orgasm, if erection be impossible—are all points for the elucidation of which we owe much to M. Lallemand and his school. But in regard to true spermatorrhœa, a flow quite passive, of semen during micturition and defecation, without sensation or even consciousness, other than its traces upon the clothes or in the urine, or on the outlet of the urethra—the same unanimity by no means exists. Indeed it is the opinion of many eminent men, among whom is Ricord, that the talented physician of Montpellier has rather injured his cause in that portion of his works which treats of true spermatorrhæa. Lallemand seems indeed to have hurried to his conclusions in rather an unphilosophical manner, and many of the means of diagnosis given by him are rather extraordinary, as coming from a man of his capacity. The true state of the case seems to be that spermatorrhœa may sometimes occur, but very rarely, and that when during defecation or micturition, traces of its presence are found, it is dependent on the fact that the sperm had remained in the ducts since the last regular pollution, the orgasm not having been sufficiently strong to throw it off. And as the prostrate is in this affection the seat of more or less morbid action, its deviations, while compressing and closing the ejaculators, afford the means of retaining the sperm until change of position or efforts shall displace them. At other times, and by far more frequently, the supposed seminal fluid is found to be merely the prostatic product pourea out in unusual quantities. The prostate may itself in certain conditions of irritation become a cause of nocturnal pollutions. But the valuable paper read before you by Dr. Frick obviates the necessity of pursuing this theme further.

Tumefaction of the prostate commonly occurs in subjects over 45 years of age, and may be partial or general. The latter is, however, comparatively rare, but occassonally is so great as to nearly fill the bladder. But even in these cases the tumefaction is not uniform, and chiefly affects the median portion. Partial tumefaction affects the body or lateral lobes. In the first case there may be a slight prominence shaded off towards the periphery, or a transverse bar, or even a pediculated tumor —that is, a projection whose base is smaller than its summit; or it may assume the opposite conditions. The lateral lobes increase either in a transverse direction or an intero-posterior one. This latter tumefaction, where unconnected with that of the body, does not much alter the urethral canal. Commonly, it simply flattens it in the form of a slit. Tumefaction of a single lobe does not alter the calibre, but changes the direction of the canal. As it increases, however, and becomes more general, the trouble does not cease with deviation, but the neck of the bladder is dragged back, and the membranous portion of the urethra pushed forwards, exerting a deplorable influence upon the bladder, and rendering catheterism difficult.

Dr. Wilkins has recently exhibited to you the proper sounds to be used in these cases, and the manner of introducing them, so that it will be unnecessary for me to reiterate them. The glandular substance is commonly found normal, sometimes softened, and the ducts enlarged and engorged; sometimes the mass is tough, with no visible alteration of structure. The results of tumefaction are hypertrophy of bladder and dilatation of the urinary passages. Unaccompanied by inflammation, these are the only serious results. It may be detected by a rectal examination The ribbon shaped faces are considered diagnostic of swelled prostate, but it is obvious that the sphincter ani alone impresses a form upon the dejections. Perhaps there is, however, reason in believing that its pressure upon the rectum may produce hemorrhoids or prolapsus of the gut. This sketch of enlarged prostrate of course extends to exceptional cases. Generally, it is not so far increased in size as to cause any great disturbance, and may be successfully treated by leeches to the perineum; warm hip baths; frictions with unguent iodine, and the catheter.

*Acute inflammation* was formerly considered of rare occurrence, but is now admitted to be frequent. According to Ricord, it is consequent upon blennorhagia and bad treatment, and is announced by perineal pain, difficult and scalding micturition, retention of urine, painful defecation, constipation, and pain along the course of the sciatic nerve, together with pain in sitting down or crossing the legs. If there be much swelling and fluctuation it will be detected by the finger; and in this examination care must be taken not to confound the apparent fluctuation of the gland with that of the vesiculæ seminales. The generative functions are commonly disordered, and nocturnal emissions may occur. The general reaction is seldom great; sometimes there is vomiting and tympanitis. The means of diagnosis between this inflammation and that of the neck of the bladder, consists in the fact that in the latter there is constant desire to pass water, while in the former the frequency of dejection does not appear to be affected. The common termination is of course in abscess, which may, if unpunctured, discharge into the urethra, destroying the ejaculatory ducts, or into the bladder, vesiculæ, the surrounding cellular tissue, rectum, or force their way into the penis and scrotum. Abscesses may be one or more in number. When they discharge into the bladder, we can only detect it by examining the urine, but when they empty into the urethra, pus is generally voided suddenly, or at intervals, in such quantities as to leave no uncertainty. The post-mortem examination shows the follicles filled with pus resembling scrofulous tubercle, the surrounding structure being healthy, or infiltrated with pus or pultaceous matter. The work of disintegration may go on so completely as to leave but a mere shell. As soon as fluctuation is ascertained, the gland should be immediately punctured. Some discussion has arisen as to the best place for doing this—many writers prefering the urethra—many the perineum. As for myself, I prefer the latter, from the much greater certainty we have in directing our instrument; and on the other hand, from the danger of producing stricture by the formation of a cicatrix in the urethra. The other part of the treatment consists in numerous leeches to the perineum, opiate enemata, and warm bath. We must also guard against retention of urine by the free use of the catheter. But in spite of our precautions, the foundation for chronic inflammation is often laid. This, when severe, presents many of the symptoms above described, which increase in violence after exposure or excess; the urine becomes forked, sometimes mingled with blood; sexual connection is painful; constipation obstinate, and great difficulty in introducing the catheter. The progress of chronic inflammation is slow, but by no means less sure on that account, with a constant tendency to complication of neighboring parts—to ulceration of the prostate; to inflammation of the bladder, and to urinary fever. At the same time the effect upon the morale is deplorable—melancholy and indifference are common, and finally the subject is carried off by exhaustion. The prognosis is, of course, unfavorable. The treatment consists of care, rest, diet, opiate clysters, hip baths, and leeches. So long as the retention of urine is but slight, the catheter must be avoided. When the necessity arrives, we are to meet it with firm measures. If we cannot insinuate the catheter through the prostate, we must perforate the gland by pushing a prepared sound through it—a much better means than opening the bladder above the pubes or per rectum.

In regard to atrophy of the prostate, calculous affections, epithelimata, cysts, tubercle, fibrous tumors, etc., I can only remark that my limits place it out of my power to give them any other consideration than a mere enumeration.

Dr. Phillip S. Field read the following paper:

The case in question is one of interest—not only from its obscurity, but also from the difference of opinion expressed by consulting physicians.

Its history (as near as I can trust to memory) is as follows: Cassandra P., an unmarried lady, formerly a resident of this county, called upon me professionally, November 21, 1856, stating that two weeks previous she contracted a violent cold by sitting in a current of air during profuse perspiration, whilst suffering with her catamenia, which caused a cessation of the same. During the night she had a severe chill, followed by high fever, and on the following morning called in a homœpathic physician, who pronounced her case to be (as she expressed herself) "dropsy of the womb." She continued under this gentleman's treatment two weeks; was then conveyed by her parents to their residence in the country, and by them I was requested to attend her. Upon examination, I found that she was laboring under *ascites*, the abdomen being enormously distended by the effusion. There was almost an entire suppression of urine, not voiding over half an ounce in twenty-four hours; which was of a brownish red color, and thickly loaded with mucus. Complained of a dull, heavy pain over the region of the kidneys, with an occasional twang, as though pierced with some sharp instrument. Cupped her freely, and ordered an infusion of juniper berries and bi. tart. potassæ. Continued this for several days, with some improvement in the secretions of the kidneys. Abdominal effusion very little if any diminished. Prescribed hydrarg. proto. chlor. ʒij; pulv. digitalis; pulv. rad. scillæ ʒi. M. and ft. Pills xx. One three times daily. Continued these until she became slightly pytalized, with marked benefit. This I followed by an occasional hydragogue cathartic, composed of elaterium and ext. colocynth comp.

Did not see her again for three weeks, owing to indisposition, during which time she was visited *once* by my friend Mr. Donaldson, who, finding her very much debilitated, suggested the propriety of giving some preparation of *iron*, and I had prepared pills of citrate of iron and quinine, each pill containing five grains—one to be taken three times daily.

On visiting her after my recovery, I found that the abdominal effusion had almost entirely disappeared, and the kidneys gradually resuming their natural secretions. She stated that during my absence, she had suffered with violent pain of an intermittent character in the lower part of the bowels, which caused me to examine the seat of pain, and discovered for the first time a hard body occupying the position of the the uterus.

I immediately suggested the necessity of making an examination per vaginam, to which she would not consent, without the advice of another physician, to which I cheerfully consented; and they selected an eminent surgeon of Baltimore, who, upon examination pronounced her pregnant. I then made an examination, found the uterus in situ; the neck somewhat enlarged, and firm. By pressing firmly the neck of the uterus I could feel a large hard substance, evidently occupying the walls of the *uterus*. I then examined the mamma and the areola around the nipple, but could discover no evidence of pregnancy. From the absence of any positive symptoms in my own judgment, I was compelled to differ with the *physician consulted*, and pronounced it a *fibrous tumor*—in which

*diagnosis* I was confirmed by the concurring opinion of Dr. Donaldson, who, by request, visited her again with me—and which *time* has more positively proved, as the lady is enjoying at this date comparatively good health. Her catamenia has been regularly performed during the last three months, though the *tumor* still remains.

[*Virginia Medical Journal.*

